# The longitudinal association between children’s growth mindset in senior primary school and their parents’ growth mindset

**DOI:** 10.3389/fpsyg.2023.1110944

**Published:** 2023-03-14

**Authors:** Jiao Chen, Chunhui Liu

**Affiliations:** ^1^Collaborative Innovation Center of Assessment Toward Basic Education Quality, Beijing Normal University, Beijing, China; ^2^PKUS Xisanqi School, Beijing, China

**Keywords:** children’s mindset, growth mindset, development trajectory, parents’ mindset, longitudinal association

## Abstract

Growth mindset plays a positive role in children’s development, but few studies use longitudinal data to investigate the developmental trajectory of children’s growth mindset. In addition, previous studies have shown that there may be no intergenerational transmission of mindset, but the influence of parents’ growth mindset on the development and change of children’s growth mindset cannot be denied. Based on the abovementioned factors, the present study used a sample of fourth-grade primary school students and their parents in Beijing (N = 4,004), and five waves of longitudinal data over two-and-a-half years were collected to identify the trajectories of growth mindset in senior primary school with latent growth modeling and to examine the effects of parents’ growth mindset with a parallel process latent growth model. The results showed the following. (1) The growth mindset of the senior primary school children decreased over time, and there were significant individual differences in the initial level and growth of mindset. (2) Children in senior primary school showed higher levels of growth mindset after two-and-a-half years if their mothers reported higher levels of growth mindset in the beginning. Children showed higher levels of growth mindset after two-and-a-half years if their mothers’ growth mindset declined slower during this period, while they showed lower levels if their mothers’ growth mindset declined rapidly; when the mothers’ growth mindset declines, the children’s growth mindset would also show a downward trend during this time. Finally, (3) there was no significant relationship between both the initial level and the decline of the father’s growth mindset and the development pattern of the children’s growth mindset.

## Introduction

Why do some people fall apart in the face of difficulties, insist that their ability is limited, and give up the challenge, while others regard failure as the mother of success and constantly pursue challenging tasks? Dweck et al. eventually developed the theory of mindset (also known as the implicit theory of intelligence) based on an “Awareness of one’s own or another’s abilities and what factors lead to the pursuit or avoidance of challenges” (Dweck and Leggett, 1988; Dweck, 1999, 2006; Blackwell et al., 2007). People who hold a fixed mindset insist that intelligence or ability is a fixed and innate trait, thus, they reject challenges beyond their control and avoid uncertain tasks. People with a growth mindset insist that ability consists of increasing knowledge and skills and that individuals can change their ability through effort. They are more likely to accept challenges because they believe that solving difficulties can improve their ability. As a quality that can promote success, a growth mindset has attracted wide attention in academic circles, and its research results have been widely applied in different practical fields, such as education and health and exert a profound impact on academic circles and society (Dweck, 2014; Alvarado et al., 2019; Yeager et al., 2019; Kaya and Karakoc, 2022).

A large number of studies have shown that a growth mindset is of vital importance to children’s development, especially in terms of academic achievement (Blackwell et al., 2007; Rattan et al., 2015; Dweck and Molden, 2017). There are also some intervention studies aimed at shaping children’s and adolescents’ growth mindsets (Yeager et al., 2019). These interventions all illustrate the variability of mindset. In addition, Dweck and Molden (2017) point out that the mindset is also theoretically variable. However, the study neglects the longitudinal change in children’s mindset with age and whether the attitude of important others in life also has a certain impact on the change in children’s mindset. Based on the aforementioned text, the current study examines developmental changes and potential predictors in children’s growth mindset to promote students’ long-term academic and other developments.

### Trajectories of children’s growth mindset

Theoretically, researchers believe that the mindset is sensitive to changes in the environment, thus, students’ mindset will change with changes in their learning environment under nonexperimental natural conditions (Dweck and Molden, 2005). In addition, some intervention studies have also found that students’ mindset changes in the expected direction in the intervention condition (Mueller and Dweck, 1998; Blackwell et al., 2007; Yeager et al., 2019, 2022). Hecht et al. (2021) presented the mindset × context framework for growth mindset interventions, and teacher belief and school climate also relate to children’s growth mindset (Yu et al., 2022). Both theoretical and empirical evidence support the variability of mindset, but few studies have explored the developmental trajectory and characteristics of students’ mindset with age, and some studies have mentioned the differences in individual mindset at different ages.

Pre-school children have not yet established the concept of stability of ability, and they give more attention to the good or bad comments that they receive in task results (Stipek and Daniels, 1990; Droege and Stipek, 1993; Ruble and Dweck, 1995). In early elementary school, children develop the concept of implicit intelligence or ability (Gunderson et al., 2013; Park et al., 2016). Kindergarten kids rated their current abilities as lower than their future abilities, fourth graders showed no difference, and fourth and eighth graders rated their intelligence as more stable than kindergarteners (Stipek and Daniels, 1990). A study of American students in grades 3 through 11 showed that when students are older, they are more likely to compare their abilities with others and think that abilities are invariable to a greater degree (Ablard and Mills, 1996). In addition, an individual’s growth mindset seems to decline during middle and high school, which leads teenagers to begin attributing their academic performance to ability rather than to effort (Harter, 2012). A study on the stability of the abilities of Chinese primary and middle school students also found that as children grow older, they tend to think that the abilities related to mathematics and music are stable and unchangeable (Cheng and Hou, 2002).

The abovementioned studies are related to the age difference in mindset in different groups. In view of the limitations of cross-sectional studies, some scholars have used longitudinal data to investigate the development of mindset. Dai and Cromley (2014) established a two-factor latent growth curve model to study the changes in entity theory and growth theory among college students. They found that students’ entity beliefs increased, while their incremental beliefs decreased over time. Then, a study on Korean high school students also used a two-factor latent growth curve model and found that both growth mindset and fixed mindset increased from eighth to tenth grade (Lee and Seo, 2019).

### Children’s growth mindset and parents’ growth mindset

Based on the importance of children’s growth mindset to their development, many studies have begun to focus on the discussion and interpretation of the predictive factors of children’s mindset. Parental education in a family is the most extensive and basic education, which is the first education received in one’s whole life (Morrow, 1995; Bandura, 1997), and parental words and actions in the process of upbringing will have a profound impact on children. Intergenerational transmission theory holds that the characteristics of parents are the root of the characteristics of children, and the characteristics of parents, such as personality, values, attitudes, cognition, and behavior, are passed on to their offspring (Bowles et al., 2009). In general, genes and the environment are the main ways in which intergenerational transmission theory occurs (Anger, 2012; Beaver and Belsky, 2012). Sociology theory has been used to demonstrate the cost transfer effect in the acquired environment (Bryant and Conger, 2002). Children acquire behaviors, values, and attitudes in their native families through observation, imitation, or training.

According to identity control theory (Burke, 1991; Burke and Stets, 2009), individuals can perceive the attitudes and behaviors of others in their environment and then compare these with their own identification standards. When others are inconsistent with themselves, individuals will adjust their behaviors and attitudes to change such inconsistencies. Parents are one of the most influential people in children’s development, and their thoughts and concepts will be transmitted to children imperceptibly in the process of parenting. When children find that parents believe that ability can be changed through more effort, they will also internalize this belief and gradually establish and develop their own belief system under the influence of parents. Parents teach by words and deeds in parent–child interactions, during which children’s perceived parental beliefs and behaviors can predict their mindset (Frank and Fabian, 2017).

Some studies have found that adults’ different views of mindset have different effects on children. For example, in a laboratory study, mothers who were induced to hold a fixed mindset showed more unconstructive involvement in their children’s learning, such as teaching children to be performance-oriented and showing controlling behaviors, which have a negative impact on children (Moorman and Pomerantz, 2010). Although the above research does not clearly show how these behaviors of parents affect children’s mindset, these behaviors do lead children to hold different mindsets (Haimovitz and Dweck, 2017). Another cross-cultural study also found a positive correlation between parents’ growth mindset and their children’s behaviors driven by growth mindset (Jose and Bellamy, 2012).

However, previous studies have not found a direct correlation between parents’ mindset and children’s mindset (Haimovitz and Dweck, 2017). For example, Haimovitz and Dweck (2016) explored what influences children’s mindset. In this cross-sectional study, 73 pairs of parents and children aged 8–12 years reported their mindset. The results showed that parents’ mindset was not related to children’s mindset.

### The present study

However, although previous research has emphasized the importance of mindset in children and adolescents and demonstrated the variability of the mindset and the possible relationship between it and parental mindset, there are still some deficiencies in these studies.

First, the current studies on the mindset of children and adolescents have mainly investigated the group differences of different age stages (Stipek and Daniels, 1990; Ablard and Mills, 1996; Cheng and Hou, 2002; Harter, 2012), and the age differences of the mindset in different studies also show inconsistency. Although these cross-sectional study designs reflected the development of children’s mindsets to some extent, it was difficult to accurately examine the developmental trajectory of individual mindsets with age. The development of the primary school is crucial for the growth of children. This stage is an important stage for children to establish their psychological characteristics, and children in senior primary school also have a clearer understanding of the concept of mindset than younger children (Stipek and MacIver, 1989). The development of a growth mindset in primary school also has implications for the future. Longitudinal research on the development and change of children’s growth mindset in senior primary school can clarify the development and change of children’s ability beliefs.

Second, the role of parental mindset in the development and change of children’s mindset cannot be denied according to intergenerational transmission theory and identity control theory. There may be two reasons regarding the relationship between parental mindset and children’s mindset not found in previous studies. One is that previous studies usually regarded parents as a whole and ignored the role of the father and mother, but there are gender differences in mindset, in which women might be more likely to hold fixed mindsets than men (Dweck and Simmons, 2014, para. 13). In addition, the roles and levels of involvement of fathers and mothers in the parenting process are different (Steinberg and Silk, 2002; Craig, 2006). The other reasons are that previous studies ignored dynamic changes in mindset. As mentioned earlier, children’s mindset changes as they grow. In addition, when combining the variability of mindset and the perspective of lifelong development, the development of individual psychology and behavior is a continuous process that covers the entire life cycle (Baltes, 1987; Baltes et al., 1998), and parental mindset may also change over time. According to family system theory (Minuchin, 1974, 1985), all family members interact with each other, thus, the development of an individual is closely related to the development of other family members.

Based on the limitations mentioned earlier in previous studies, this study was intended to be based on longitudinal data to explore the following two questions: (1) What is the developmental trajectory of the growth mindset of fathers, mothers, and children separately? (2) How do the developmental changes in fathers’ and mother’s growth mindset affect their children’s growth mindset?

For the first question, it was expected that children’s growth mindset would decrease on average, as previous research has provided evidence that older students are more likely to believe that ability is immutable (Stipek and Daniels, 1990; Ablard and Mills, 1996; Cheng and Hou, 2002). Regarding the developmental direction of the parental growth mindset, we did not make any assumptions. Regarding the second question, because parents influence the developmental outcomes of their children, we were interested in the long-term effects of parental growth mindset on their children’s growth mindset. The first wave (children in fourth grade, spring semester) of measurement was chosen as the reference point of fathers’ and mothers’ growth mindset, while the last wave of measurement was chosen as the reference point of children’s growth mindset, which means that the intercept refers to the level of children’s growth mindset in the spring semester of sixth grade. We expected that fathers’ and mothers’ initial growth mindset and their rate of change would positively predict children’s growth mindset in the last wave of measurement and its change.

In previous studies, socioeconomic status (SES) has been found to be a crucial factor in mindset (Mesmin et al., 2019). In addition, there are significant age differences and gender differences in mindset (Stipek and MacIver, 1989; Diseth et al., 2014; Gunderson et al., 2017a). Therefore, students’ SES, age, and gender were taken as the control variables in this study. Specifically, children’s gender factors play an important role in their development, and there have been many studies on the differences between boys and girls in the parenting process (Siegal, 1987; Lytton and Romney, 1991; Shelly, 2005). For example, mothers engage in personal parenting more frequently with their daughters than with their sons, and fathers have relatively less involvement with their daughters than with their sons (Michelle and Charles, 2008). Given the different effects of parental beliefs and behaviors on boys and girls, we also explored gender differences in the relationship between parental growth mindset and children’s growth mindset.

## Methods

### Participants and procedure

The participants in this study came from a large longitudinal study (Child Academic and Psychological Development Study-CAPS) that examined the influence of family, school, and individual factors on children’s academic and mental health development. The project began in the fall term of 2016 (the fall semester of the fourth grade), and each test interval is half a year. The data used in the current study were gathered in five waves (the five waves were given in the spring semester of the fourth grade, the fall semester of the fifth grade, the spring semester of the fifth grade, the fall semester of the sixth grade, and the spring semester of the sixth grade) from children and their parents (both mothers and fathers) of 36 public primary schools in Baoding City, Hebei Province, China. The economic level of Hebei Province was slightly lower than the national average level, with a national *per capita* disposable income of 25,973.8 yuan for the national average and 21,484.1 yuan for Hebei Province in 2017 (National Bureau of Statistics of China, 2018).

The class was regarded as a unit for the test at each time point in the students’ tests. The trained supervisors (graduate students and undergraduate students) read the uniform instruction and told the students that the test was only for scientific research, the teachers and parents could not see their answers, and the questionnaires would be collected immediately after the test. The parents’ questionnaire and informed consent were brought home by the students the day before the test. After the parents agreed to participate in the test, they answered the father’s or mother’s questionnaire independently, and data were collected the next day. These procedures were approved by the Institutional Review Board of the Collaborative Innovation Center of Assessment toward Basic Education Quality, Beijing Normal University.

A total of 4,004 children were enrolled in the initial sample, and questionnaires with a miss rate of more than one in 10 were removed; therefore, 3,798 children were involved in the study at Time 1. Due to the transfer of students, sick leave, or other reasons, there was a certain loss in the data. The final sample included 3,627 children who had completed at least three tests, among whom 1,885 were boys (52%) and 1,742 were girls (48%), with an average age of 10.421 years (SD = 0.518) at Time 1. We examined whether systematic differences were observable in the children involved and those who dropped out of the study. Chi-square tests revealed no differences between the two groups in the core variable of growth mindset at Time 1, *χ^2^*(1) = 1.284, *p* = 0.257, children’s gender, *χ^2^*(1) = 0.928, *p* = 0.336, children’s age, *χ^2^*(1) = 0.256, *p* = 0.613, and annual household income, *χ^2^*(1) = 3.430, *p* = 0.064. However, there were significant differences in mothers’ education level, *χ^2^*(1) = 27.116, *p* < 0.001, and fathers’ education level, *χ^2^*(1) = 34.717, *p* < 0.001. Little’s missing completely at random test indicated that missing data were consistent with the pattern of the missing completely at random test (*χ^2^* = 34.912, *p* = 0.424). Among these students, their parents also had to complete three or more tests, and 3,436 fathers and 3,509 mothers were included in the analysis. Nearly 14.3% of fathers and 19.2% of mothers completed primary school education or below, 67.1% of fathers and 60.7% of mothers completed middle school education or secondary vocational school education, 11.6% of fathers and 12.5% of mothers completed junior high school education or technical secondary school education, and the remaining parents completed a 3-year college or bachelor’s degree or higher.

### Measures

#### Growth mindset

Growth mindset was independently self-reported by children, their fathers, and their mothers at five time points by using the Mindset subscale, which was originally developed by Dweck (1999). The original scale contains 8 items, and we used four of these questions to measure the growth mindset. An example item was “Everyone can significantly change his or her ability.” The questions were graded on a 5-point scale, with 1 for “strongly disagree” and 5 for “strongly agree.” Higher scores indicated a higher growth mindset. The Cronbach’s alpha coefficients of the five tests ranged from 0.704 to 0.811 for children, from 0.765 to 0.827 for fathers, and from 0.744 to 0.836 for mothers.

#### Demographics

In this study, the school provided the children’s gender and birth date of the students. Parents reported their education level and family annual income at time point 1. The average annual household income reported by the father and mother was taken as the annual household income. The education level of the parents was coded as follows: (1) primary school and below; (2) middle school education or secondary vocational school; (3) junior high school or technical secondary school; (4) 3-year college; (5) a bachelor’s degree; and (6) a master’s degree or above. Family annual income was coded as follows: (1) less than 3,600 yuan; (2) 3,601–7,200 yuan; (3) 7,201–14,000 yuan; (4) 14,001–30,000 yuan; (5) 30,001–50,000 yuan; (6) 50,001–100,000 yuan; (7) 100,001–200,000 yuan; (8) 200,001–300,000 yuan; (9) 300,001–500,000 yuan; and (10) more than 500,001 yuan. Therefore, in this study, the average father’s education level was 2.14 (SD = 0.813), the average mother’s education level was 2.12 (SD = 0.880), and the average family annual income was 4.063 (SD = 1.863).

### Data analytic plan

First, SPSS version 21.0 was used to conduct the descriptive statistics on the research variables and calculate the correlation coefficient to observe not only the relationship between parents’ growth mindset and children’s growth mindset but also the stability of their growth mindset. To test whether the change in growth mindset was due to true changes in the participants’ beliefs rather than the change in the scale structure, we performed a longitudinal measurement invariance analysis. The configuration invariance model, weak invariance model, and strong invariance model were established in turn, and then, these nested models were compared by using the chi-square difference test. If the chi-square difference test between the two models is significant, then the measurement invariance is not satisfied (Satorra and Bentler, 2001). However, previous studies have found that the chi-square test is easily affected by the sample size; with an increase in the sample size, even a small difference will result in a significant difference, thus, the Fitting Index Difference method can also be used to test measurement invariance (Cheung and Rensvold, 2002; Meade et al., 2008).

To examine the mean growth pattern in the growth mindset for children, fathers, and mothers, we used Mplus version 7.11 (Muthén et al., 2013) to establish three separate univariate latent growth curve models to investigate the initial level and development trend of the growth mindset and whether there are individual differences. There were two latent factors, namely, an intercept factor (which described the average initial level) and a slope factor (which described the development and change). The initial level (intercept) and growth (slope) were allowed to correlate. The load on the intercept factor of the repeated measurement was fixed at 1 at five time points. As we were concerned about the long-term impact of parental mindset on children’s mindset, the load on the slope was fixed at 0, 1, 2, 3, and 4 according to the time interval of the test for fathers and mothers from T1 to T5, while it was fixed at −4, −3, −2, −1, and 0 for children from T1 to T5 (Biesanz et al., 2004). Moreover, the variance of the intercept and slope described the variability of the initial level and change separately.

**Figure 1:**
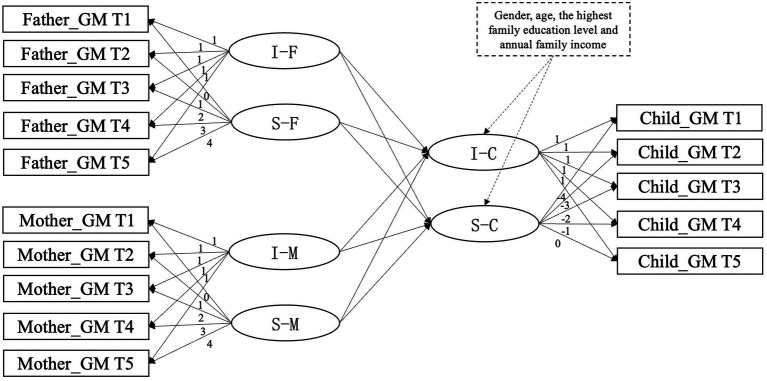
Parallel process latent growth curve model of children’s and father’s and mother’s growth mindset. For clarity reasons, intercept and slope factor loadings for variable and correlation coefficients are not depicted. GM, growth mindset; S, slope.

Then, a parallel process latent growth curve model was established by using the abovementioned models to examine the associations between developmental changes in growth mindset for children and their fathers and mothers. In this model, the latent factors (intercept and/or slope) of parental growth mindset were used to predict the latent factors (intercept and/or slope) of children’s growth mindset while controlling for the correlation between parent–child’s growth mindset in the same period. We established two models with or without control variables. Children’s gender and age, father’s education level, mother’s education level, and annual family income were added to the conditional parallel process latent growth model (Figure 1).

Finally, to test whether the paths from parents’ growth mindset to children’s growth mindset differed by children’s gender, we conducted multiple group analyses. Specifically, we divided boys and girls into two groups and performed a series of Wald tests for different paths from parents’ growth mindset to children’s growth mindset.

In this study, model fit was assessed by the comparative fit index (CFI) and root mean square error of approximation (RMSEA; Hu and Bentler, 1999). A maximum likelihood estimation (ML) was used for model estimation, and the full-information maximum likelihood method (FIML) was used for missing data processing.

## Results

### Preliminary analyses

Table 1 shows the mean, standard deviation, and correlation coefficient of the primary variables. According to the results in Table 1, the correlation for children’s growth mindset at the five time points was between 0.243 and 0.492; for fathers’ growth mindset, it was between 0.195 and 0.334; and for mothers’ growth mindset, it was between 0.208 and 0.467, which indicates that their growth mindset had a moderate degree of stability. In addition, the children’s growth mindset scores were positively correlated with the fathers’ scores (*r*’s = 0.056–0.195) and mothers’ scores (*r*’s = 0.057–0.179). In general, the parent–child correlation was smaller than the intra-individual correlation.

**Table 1:** Descriptive statistics and Pearson correlations for the main study variables.

	1	2	3	4	5	6	7	8	9	10	11	12	13	14	15
M	3.397	3.418	3.495	3.352	3.368	3.062	3.007	3.010	2.969	2.986	3.038	2.982	2.984	2.976	2.966
SD	0.838	0.857	0.849	0.894	0.898	0.749	0.766	0.754	0.774	0.800	0.732	0.736	0.748	0.756	0.785
1. Child GM T1	1														
2. Child GM T2	0.339^**^	1													
3. Child GM T3	0.311^**^	0.400^**^	1												
4. Child GM T4	0.243^**^	0.374^**^	0.460^**^	1											
5. Child GM T5	0.247^**^	0.349^**^	0.430^**^	0.492^**^	1										
6. Father GM T1	0.155^**^	0.076^**^	0.068^**^	0.060^**^	0.056^**^	1									
7. Father GM T2	0.099^**^	0.195^**^	0.115^**^	0.118^**^	0.070^**^	0.228^**^	1								
8. Father GM T3	0.099^**^	0.143^**^	0.179^**^	0.115^**^	0.087^**^	0.234^**^	0.313^**^	1							
9. Father GM T4	0.104^**^	0.115^**^	0.128^**^	0.158^**^	0.096^**^	0.196^**^	0.279^**^	0.275^**^	1						
10. Father GM T5	0.095^**^	0.136^**^	0.134^**^	0.150^**^	0.172^**^	0.195^**^	0.284^**^	0.280^**^	0.334^**^	1					
11. Mother GM T1	0.174^**^	0.110^**^	0.084^**^	0.064^**^	0.067^**^	0.419^**^	0.237^**^	0.203^**^	0.199^**^	0.208^**^	1				
12. Mother GM T2	0.105^**^	0.179^**^	0.142^**^	0.119^**^	0.090^**^	0.199^**^	0.451^**^	0.225^**^	0.223^**^	0.206^**^	0.352^**^	1			
13. Mother GM T3	0.113^**^	0.131^**^	0.181^**^	0.127^**^	0.094^**^	0.186^**^	0.242^**^	0.451^**^	0.232^**^	0.239^**^	0.298^**^	0.344^**^	1		
14. Mother GM T4	0.057^**^	0.107^**^	0.133^**^	0.172^**^	0.092^**^	0.177^**^	0.233^**^	0.250^**^	0.472^**^	0.239^**^	0.304^**^	0.320^**^	0.363^**^	1	
15. Mother GM T5	0.083^**^	0.128^**^	0.139^**^	0.159^**^	0.184^**^	0.180^**^	0.206^**^	0.214^**^	0.269^**^	0.467^**^	0.278^**^	0.304^**^	0.325^**^	0.380^**^	1

GM, growth mindset; T1, Time 1; T2, Time 2; T3, Time 3; T4, Time 4; T5, Time 5. ^*^p < 0.05, ^**^p < 0.01, ^***^p < 0.01.

**Table 2:** Longitudinal confirmatory factor analysis on growth mindset.

Model	*χ^2^*	df	CFI	TLI	RMSEA	Δ*χ^2^*	Δdf	*p*
**Child GM**								
Configuration invariance model	914.577	155	0.967	0.960	0.037			
Weak invariance model	931.768	167	0.967	0.963	0.036	17.191	12	>0.05
**Father GM**								
Configuration invariance model	787.170	155	0.973	0.967	0.034			
Weak invariance model	791.383	167	0.973	0.970	0.032	4.213	12	>0.05
**Mother GM**								
Configuration invariance model	952.833	155	0.967	0.960	0.038			
Weak invariance model	967.304	165	0.967	0.962	0.037	14.471	10	>0.05

GM, growth mindset. ^*^p < 0.05, ^**^p < 0.01, ^***^p < 0.01.

A longitudinal measurement invariance analysis for children’s growth mindset showed that the configuration invariance model fit well, and the weak invariance model also fit well. Comparing these two models, Δ*χ^2^* = 17.191, Δdf = 12, *p* > 0.05, ΔCFI = 0 < 0.01, ΔTLI = 0.003 < 0.01, the weak invariance model was accepted. Then, comparing the weak invariance model and strong invariance model, Δ*χ^2^* = 40.053, Δdf = 12, *p* < 0.05, ΔCFI = 0.001 < 0.01, ΔTLI = 0.001 < 0.01, we showed that strong measurement invariance was satisfied. The results suggested that the structure of children’s growth mindset remained invariant of the factor loadings and intercepts across the five time points. The measurement invariance of fathers’ growth mindset and mothers’ growth mindset was similar to that of children’s (for specific parameters, refer to Table 2).

### Univariate growth trajectories

**Figure 2:**
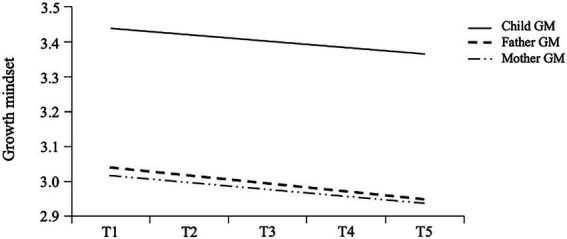
The developmental trajectory of growth mindset. GM, growth mindset.

The model fit for the univariate growth trajectory of growth mindset was excellent for children/fathers/mothers, and the parameter estimates are shown in Table 3. Figure 2 shows the developmental trajectory of growth mindset. In the unconditional latent growth curve model for the children, the initial level of the growth mindset was 3.431 (*p* < 0.001); that is, the mean of the children’s growth mindset was 3.431 at the fifth measurement and showed a linear downward trend over time at the five time points of the test, with a slope of −0.011 (*p* = 0.008). The correlation coefficient between the intercept and the slope was r = −0.015, *p* < 0.001, which indicated that the children’s growth mindset at T5 was inversely related to the rates of change over time. In addition, both the intercept variance (*σ^2^* = 0.262, *p* < 0.001) and the slope variance (*σ^2^* = 0.021, *p* < 0.01) were significant, which indicated significant individual differences in both the initial level and the change in the growth mindset of senior primary school children. The parental unconditional latent growth curve models were similar to the children’s model, and the slope factor of growth mindset was significant and negative (*b* = −0.019, *p* < 0.01, for fathers, *b* = −0.016, *p* < 0.01, for mothers). Significant intercept and slope variance indicated significant individual differences in starting levels and rates of change in fathers’ and mothers’ growth mindset. However, there was a statistically significant and negative correlation between the intercept and slope in the mothers’ model but not in the fathers’ model.

**Table 3:** Parameter estimates and model fit for univariate growth models.

Variable	β	b(SE)	*p*	Model fit				
				*χ^2^*/(df)	*p*	CFI	TLI	RMSEA
**Child GM**				125.075(10)	<0.001	0.966	0.966	0.056
Means
Intercept	6.703	3.431(0.012)	<0.001					
Linear slope	−0.078	−0.011(0.004)	0.008					
Variances
Intercept	1	0.262(0.014)	<0.001					
Linear slope	1	0.021(0.002)	<0.001					
Correlation
Intercept with slope	−0.209	−0.015(0.004)	<0.001					
**Father GM**				33.512 (10)	<0.001	0.985	0.985	0.026
Means
Intercept	8.031	3.046(0.011)	<0.001					
Linear slope	−0.223	−0.019(0.004)	<0.001					
Variances
Intercept	1	0.144(0.012)	<0.001					
Linear slope	1	0.007(0.002)	<0.001					
Correlation
Intercept with slope	−0.115	−0.004(0.004)	0.324					
**Mother GM**				17.801 (10)	0.058	0.997	0.997	0.015
Means
Intercept	6.843	3.022(0.011)	<0.001					
Linear slope	−0.174	−0.016(0.004)	<0.001					
Variances
Intercept	1	0.195(0.012)	<0.001					
Linear slope	1	0.008(0.001)	<0.001					
Correlation
Intercept with slope	−0.249	−0.010(0.003)	0.004					

GM, growth mindset. ^*^p < 0.05, ^**^p < 0.01, ^***^p < 0.01.

### Parallel process latent growth curve model

Then, a parallel process latent growth curve model was established that included the trajectory of children’s, fathers’, and mothers’ growth mindset. First, the three univariate growth curve models mentioned earlier were combined within one parallel process latent growth curve model. This unconditional model indicated an adequate fit, *χ^2^(78)* = 225.761, *p* < 0.001, CFI = 0.986, TLI = 0.981, RSMEA = 0.023. Then, a conditional parallel process latent growth curve model was established that controlled for children’s gender and age, father’s education level, mother’s education level, and annual family income. This conditional model also fits the data well, *χ^2^(143)* = 290.002, *p* < 0.001, CFI = 0.986, TLI = 0.982, RSMEA = 0.017. The parameter estimates are reported in Table 4. In the conditional model, children’s gender was positively associated with the intercept of children’s growth mindset (β = −0.008, *p* < 0.01), while there was no significant correlation among children’s age, father’s education level, mother’s education level, annual family income, and the intercept/slope of children’s growth mindset.

**Table 4:** Parameter estimates for parallel process latent growth curve model.

Path	β	b(SE)	*p*
Unconditional model
I_child_GM on I_father_GM	0.105	0.189 (0.120)	0.114
I_child_GM on S_father_GM	0.160	1.317 (0.739)	0.075
I_child_GM on I_mother_GM	0.127	0.197 (0.091)	**0.030**
I_child_GM on S_mother_GM	0.215	1.658 (0.624)	**0.008**
S_child_GM on I_father_GM	−0.056	−0.021 (0.040)	0.598
S_child_GM on S_father_GM	0.009	0.016 (0.241)	0.946
S_child_GM on I_mother_GM	0.005	0.002 (0.030)	0.960
S_child_GM on S_mother_GM	0.362	0.585 (0.212)	**0.006**
Conditional model
I_child_GM on I_father_GM	0.123	0.220 (0.120)	0.066
I_child_GM on S_father_GM	0.134	1.084 (0.708)	0.126
I_child_GM on I_mother_GM	0.109	0.169 (0.091)	0.064
I_child_GM on S_mother_GM	0.227	1.822 (0.675)	**0.007**
S_child_GM on I_father_GM	−0.068	−0.026 (0.040)	0.525
S_child_GM on S_father_GM	0.021	0.036 (0.235)	0.879
S_child_GM on I_mother_GM	0.023	0.007 (0.030)	0.810
S_child_GM on S_mother_GM	0.369	0.617 (0.230)	**0.007**

GM, growth mindset. ^*^p < 0.05, ^**^p < 0.01, ^***^p < 0.01. The bold values are the significant paths in the Parallel process latent growth curve model.

An unconditional model showed that the initial level of maternal growth mindset could positively predict the initial level of children’s growth mindset (*β* = 0.127, *p* = 0.03), which means that maternal growth mindset at Time 1 positively predicts children’s growth mindset at Time 5. However, this path was marginally significant after controlling for demographic variables (*β* = 0.109, *p* = 0 0.064) in the conditional model. Moreover, the paths from the slope of maternal growth mindset to children’s intercept were both significant in the conditional (*β* = 0.215, *p* = 0.008) and unconditional models (*β* = 0.227, *p* = 0.007), which indicates that the change in mothers’ growth mindset can influence the level of children’s growth mindset in the fifth test. The results also showed a significant and positive link from the slope of maternal growth mindset to children’s slope in the conditional (*β* = 0.362, *p* = 0.006) and unconditional models (*β* = 0.369, *p* = 0.007); that is, when the mothers’ growth mindset declined more rapidly, the children’s growth mindset declined more rapidly. In contrast, neither the intercept nor the slope of fathers’ growth mindset predicted the change in children’s growth mindset or children’s growth mindset at Time 5.

### Multiple group analyses

Finally, we tested whether any of the paths from the latent growth factors of fathers’ or mothers’ growth mindset to the latent growth factors of children’s growth mindset differed by children’s gender. Eight paths were checked by the Wald test (refer to Table 4). The results showed that only one path test was significant, that is, the path from the intercept of fathers’ growth mindset to the intercept of children’s growth mindset differed by children’s gender [*χ^2^(1)* = 4.154, *p* < 0.042]. Specifically, paternal growth mindset in the first test predicted girls’ growth mindset in the fifth test (*β* = 0.253, *p* = 0.014) but not boys’ growth mindset (*β* = −0.038, *p* = 0.709).

## Discussion

Mindset has a powerful impact on many aspects of child development, such as educational attainment, emotions, health, and self-esteem (Robins and Pals, 2002; Blackwell et al., 2007; Yeager et al., 2011, 2013, 2014; Romero et al., 2014). However, less is known about the trajectory of mindset in children or the role of parents’ mindset in shaping children’s mindset over a long period of time and its growth pattern. In this study, through five waves of longitudinal data, latent variable growth models were adopted to explore these questions. The results showed that the growth mindset of the senior primary school children decreased over time, and there were significant individual differences in the initial level and growth of mindset. Paternal and maternal growth mindsets were also on the decline. Our results in the parallel process latent growth curve model suggested that whether controlling for children’s gender and age, paternal education level, maternal education level, and annual family income (1) children in senior primary school showed higher levels of growth mindset after two-and-a-half years if their mothers reported higher levels of growth mindset in the beginning; (2) children showed higher levels of growth mindset after two-and-a-half years if their mothers’ growth mindset declined slower during this period, while they showed lower levels if their mothers’ growth mindset declined rapidly; (3) the mothers’ growth mindset declined and the children’s growth mindset also showed a downward trend during this time; and (4) there was no significant relationship between both the initial level and the decline of the father’s mindset and the development pattern of the children’s mindset.

### The developmental trajectory and characteristics of growth mindset

Consistent with the results of previous studies (Ablard and Mills, 1996; Cheng and Hou, 2002; Harter, 2012), in this study, the growth mindset of children in senior primary school showed a downward trend. With increasing age, the children at this age increasingly believed that they were invariable and could not be improved through tasks. The developmental trajectory of children’s growth mindset is consistent with the development of children’s average levels of self-evaluation. In elementary school, children’s social cognition changes in the following aspects. One is that children can gradually distinguish between their ideal selves and their real selves, and they do not see themselves as positively as they did when they were young (Harter, 2006b). Another aspect is that they acquire increasingly more social information, which they use to make comparisons when evaluating themselves (Harter, 2006b; Gore and Cross, 2014). Another aspect is that children gradually learn to put themselves in others’ shoes, and they know how others evaluate themselves, which is not always positive (Harter, 2006a). Accordingly, because of the improvement in social cognition, children’s self-evaluation will decline (Robins and Pals, 2002; Harter, 2006b,c). Reduced self-evaluation permeates everything, with children acting less confident, refusing challenges to avoid failure, and not believing that their abilities are malleable, which is the decline of growth mindset. In addition, students in the upper grades of primary school are about to enter adolescence, face challenges about biological, cognitive, emotional, and social changes, and become more negative in early adolescence, particularly in the domains of academic abilities and self-expression (Eccles et al., 1984; Marsh, 1989; Wigfield et al., 1991).

In our study, parents’ mindset of children in senior primary school also decreased over time. People in middle age, after experiencing the struggles of career and life, become more mature and stable psychologically and emotionally. Studies have shown that adults experience both job burnout and parenting burnout (Mikolajczak et al., 2019; Roskam and Mikolajczak, 2020). This relatively less positive attitude and emotion can spill over into every aspect of life. Based on this view, parents may think that hard work is less likely to change their ability.

In this study, we found that the initial level and rate of descent of the growth mindset had a significantly negative correlation in both children and mothers but not fathers, that is, when the level of mindset is higher, the rate of decline is faster. This may reflect the “regression-toward-the-mean” effect. Both the initial level and the rate of change of mindset showed significant individual differences in all the participants, which is the same as in previous studies (Bempechat et al., 1991; Pomerantz and Ruble, 1997; Wang, 2003). There are many reasons for this difference, including not only the characteristics of the participants themselves but also the characteristics of their significant others, and even the influence of the family’s socioemotional environment could affect children’s beliefs directly and indirectly (Simpkins et al., 2015). Therefore, this explains the importance of exploring the antecedent variables that influence the development of different age groups’ mindsets.

### The influence of parents’ growth mindset on the development of children’s growth mindset in senior primary school

In the process of parental education involvement, according to self-determination theory, parents support their children in three ways, namely, affinity support or engagement (respect and warmth); ability support (adequate help and noncritical feedback); and autonomy support (providing choices and encouraging exploration; Grolnick et al., 1997; Joussemet et al., 2008). Competence support refers to parents helping their children build a sense of ability or competence and giving attention to learning and developing existing and new skills, such as discovering their children’s gifts and interests and providing support for developing these gifts and interests. In the process of cultivating children’s sense of ability, children form an understanding of their own or others’ abilities and gradually establish a concept of ability and stability of ability. Parents with a growth mindset insist that ability consists of increasing knowledge and skills, and they will devote more attention to the learning and development of children’s knowledge and skills in parent–child interactions, which may influence children’s mindset from the two aspects of parenting beliefs and behavioral interaction. In terms of parenting beliefs, parents’ belief in their children’s failure (failure mindset)—Do parents view their children’s failures as positive and helpful or do they view these failures negatively as obstacles to their children’s success?—will affect children’s mindset. Parents with a growth mindset are more likely to look at failure positively, let the child learn from the failure of growth, help the child to grow in the failure and to not be afraid of challenges, and believe that efforts will be made to help the child to form a growth mindset (Haimovitz and Dweck, 2016). In terms of behavioral interaction, a previous study with an online sample of 300 parents indicated that when parents believed more that their abilities were fixed, they were more likely to engage in controlling and performance-oriented behaviors; in contrast, parents engaged in autonomy-supportive and mastery-oriented behaviors when they believed that their abilities were not fixed (Muenks et al., 2015). In addition, the way that parents praise and criticize their children can also have an impact on their children’s mindset. Person praise, which attributes a child’s success to a fixed trait, such as praising children for cleverness, will build the perception that a child’s abilities are beyond his or her control. Process praise and criticism, which attributes the success and failure of the child to his efforts to give more or less, will make the child think that he can achieve success through his own efforts and form a growth mindset (Xing et al., 2011; Gunderson et al., 2017b, 2018). Parents with a growth mindset believe that ability is malleable, thus, praise and criticism for children are more based on the external performance of children and less evaluated from the fixed characteristics of children, which is also conducive to the formation and development of children’s mindset. This study indicates that mothers’ mindset plays a vital role in the development and change of children’s mindset.

However, we did not find an influence on fathers’ mindset; this is not to say that fathers have no influence on all children samples, although parents play different roles in the care of their children. Mothers give more attention to the physical development of their children, while fathers exert an important influence on their children’s intelligence, emotional volition, socialization, and other aspects through interaction with their children (Steinberg and Silk, 2002; Paquette, 2004; Craig, 2006; Xu and Li, 2019). In general, mothers assume the primary role of child care, and during the middle and teenage years, mothers seem to interact with their children more frequently than fathers (especially regarding care and daily family tasks) by spending significantly more time with their children than fathers and other family members (Craig and Mullan, 2011). Moreover, considering traditional Confucianism in China, the idea that fathers are mainly responsible for work and mothers are mainly responsible for the family is deeply rooted (Wu et al., 2012). Even the role of the mother may restrict the involvement of the father in the education of the child, which is called maternal gatekeeping (Allen and Hawkins, 1999; Zvara et al., 2013), thus, the mother plays a greater role in the education of the child. The beliefs and values of the mother will be transferred to the child in more frequent parent–child interactions, and the child’s perception of the mother’s beliefs will be greater than that of other caregivers, thus, it is speculated that the formation and development of the child’s mindset will be more influenced by the mother.

Multiple group analyses suggested that fathers’ mindset influences girls’ mindset more than boys’ mindset. This is inconsistent with the results of previous studies that supported the idea that children may be more likely to model the attitudes, values, and behaviors of their same-gender parent (Bussey and Bandura, 2004; Pomerantz et al., 2004). The probable reason is that fathers tend to be more democratic with their daughters than with their sons (Kausar and Shafique, 2008), and fathers dote on their daughters and give them more support. In addition, girls show more attachment and identification with their fathers because of the Oedipus complex.

### Implications and limitations

In general, this study investigated the developmental trajectory of children’s mindsets, and there were significant individual differences in the initial level and rising rate of children’s mindsets in senior primary school. Previous studies have not found a link between parental mindset and children’s mindset, while the relationship was proven from the perspective of the developmental trajectory, and we also found the role of fathers and mothers to be different. Theoretically, this study enriches the longitudinal research on mindset and provides support for mindset interventions and a reference for research on the parent–child mindset. From the perspective of practice, this study indicates the different roles of parents in the development of children and suggests that parents should actively participate in family education.

Although this study extends prior studies in this area, there are still some limitations to note. First, we investigate the relationship between the mindset of children in the upper grade of primary school and their parents’ mindset in the Chinese context; however, the generalizability of the current study to other populations is less clear. For example, it is well known that as adolescents transition into adolescence, they are faced with greater environmental changes, and how their mindset develops and is influenced by other factors during this process remains to be further investigated. Future studies could also investigate the relationship that we found in this study in other cultural contexts. Second, this study does not explore the specific mechanism of the influence of parental mindset on the development of children’s mindset, and future studies could investigate this mechanism in more detail so that more parents can understand this relationship and give educational advice specifically and operationally.

## Data availability statement

The raw data supporting the conclusions of this article will be made available by the authors, without undue reservation.

## Ethics statement

This study was carried out in accordance with the recommendations of the Institutional Review Board of the Collaborative Innovation Center of Assessment toward Basic Education Quality, Beijing Normal University. Written informed consent to participate in this study was provided by the participants or their legal guardian before evaluation.

## Author contributions

JC conceived and designed the study, analyzed and interpreted the data, and wrote and revised the manuscript. CL provided conceptual and written feedback on the manuscript. All authors contributed to the article and approved the submitted version.

## Funding

This study was supported by the 2017 Project for Youth of Beijing Education Science 13th Five-Year Plan (The relationship between parents’ failure mindsets and pupils ‘mindsets: a longitudinal study, project number BCEA17047).

## Conflict of interest

The authors declare that the research was conducted in the absence of any commercial or financial relationships that could be construed as a potential conflict of interest.

## Publisher’s note

All claims expressed in this article are solely those of the authors and do not necessarily represent those of their affiliated organizations, or those of the publisher, the editors and the reviewers. Any product that may be evaluated in this article, or claim that may be made by its manufacturer, is not guaranteed or endorsed by the publisher.
